# Quantitative Eye Gaze and Movement Differences in Visuomotor Adaptations to Varying Task Demands Among Upper-Extremity Prosthesis Users

**DOI:** 10.1001/jamanetworkopen.2019.11197

**Published:** 2019-09-13

**Authors:** Jacqueline S. Hebert, Quinn A. Boser, Aïda M. Valevicius, Hiroki Tanikawa, Ewen B. Lavoie, Albert H. Vette, Patrick M. Pilarski, Craig S. Chapman

**Affiliations:** 1 Department of Medicine, Faculty of Medicine and Dentistry, University of Alberta, Edmonton, Alberta, Canada; 2 Department of Biomedical Engineering, University of Alberta, Edmonton, Alberta, Canada; 3 Faculty of Rehabilitation, School of Health Sciences, Fujita Health University, Toyoake, Aichi, Japan; 4 Faculty of Kinesiology, Sport, and Recreation, University of Alberta, Edmonton, Alberta, Canada; 5 Department of Mechanical Engineering, Faculty of Engineering, University of Alberta, Edmonton, Alberta, Canada; 6 Department of Computing Science, University of Alberta, Edmonton, Alberta, Canada

## Abstract

**Importance:**

New treatments for upper-limb amputation aim to improve movement quality and reduce visual attention to the prosthesis. However, evaluation is limited by a lack of understanding of the essential features of human-prosthesis behavior and by an absence of consistent task protocols.

**Objective:**

To evaluate whether task selection is a factor in visuomotor adaptations by prosthesis users to accomplish 2 tasks easily performed by individuals with normal arm function.

**Design, Setting, and Participants:**

This cross-sectional study was conducted in a single research center at the University of Alberta, Edmonton, Alberta, Canada. Upper-extremity prosthesis users were recruited from January 1, 2016, through December 31, 2016, and individuals with normal arm function were recruited from October 1, 2015, through November 30, 2015. Eight prosthesis users and 16 participants with normal arm function were asked to perform 2 goal-directed tasks with synchronized motion capture and eye tracking. Data analysis was performed from December 3, 2018, to April 15, 2019.

**Main Outcome and Measures:**

Movement time, eye fixation, and range of motion of the upper body during 2 object transfer tasks (cup and box) were the main outcomes.

**Results:**

A convenience sample comprised 8 male prosthesis users with acquired amputation (mean [range] age, 45 [30-64] years), along with 16 participants with normal arm function (8 [50%] of whom were men; mean [range] age, 26 [18-43] years; mean [range] height, 172.3 [158.0-186.0] cm; all right handed). Prosthesis users spent a disproportionately prolonged mean (SD) time in grasp and release phases when handling the cups (grasp: 2.0 [2.3] seconds vs 0.9 [0.8] seconds; *P* < .001; release: 1.1 [0.6] seconds vs 0.7 [0.4] seconds; *P* < .001). Prosthesis users also had increased mean (SD) visual fixations on the hand for the cup compared with the box task during reach (10.2% [12.1%] vs 2.2% [2.8%]) and transport (37.1% [9.7%] vs 22.3% [7.6%]). Fixations on the hand for both tasks were significantly greater for prosthesis users compared with normative values. Prosthesis users had significantly more trunk flexion and extension for the box task compared with the cup task (mean [SD] trunk range of motion, 32.1 [10.7] degrees vs 21.2 [3.7] degrees; *P* = .01), with all trunk motions greater than normative values. The box task required greater shoulder movements compared with the cup task for prosthesis users (mean [SD] flexion and extension; 51.3 [12.6] degrees vs 41.0 [9.4] degrees, *P* = .01; abduction and adduction: 40.5 [7.2] degrees vs 32.3 [5.1] degrees, *P* = .02; rotation: 50.6 [15.7] degrees vs 35.5 [10.0] degrees, *P* = .02). However, other than shoulder abduction and adduction for the box task, these values were less than those seen for participants with normal arm function.

**Conclusions and Relevance:**

This study suggests that prosthesis users have an inherently different way of adapting to varying task demands, therefore suggesting that task selection is crucial in evaluating visuomotor performance. The cup task required greater compensatory visual fixations and prolonged grasp and release movements, and the box task required specific kinematic compensatory strategies as well as increased visual fixation. This is the first study to date to examine visuomotor differences in prosthesis users across varying task demands, and the findings appear to highlight the advantages of quantitative assessment in understanding human-prosthesis interaction.

## Key Points

**Question:**

Is task selection a factor in visuomotor adaptation strategies and therefore a complication in measuring outcomes for users of upper-extremity prostheses?

**Findings:**

In this cross-sectional study of 8 prosthesis users and 16 participants with normal arm function, between tasks, prosthesis users changed their visuomotor compensatory strategies, and these strategies were different from those used by participants with normal arm function when performing the tasks. However, for a given task, prosthesis users demonstrated similar compensations despite varying amputation levels and technology.

**Meaning:**

This study suggests that prosthesis users have inherently different ways of functioning for object interaction tasks compared with individuals with normal arm function, and compensation strategies appear to vary depending on the task.

## Teaser

This cross-sectional study examines whether task selection is a factor in visuomotor adaptation strategies to perform goal-directed object movement tasks in individuals with an upper-limb prosthesis compared with individuals with normal arm function.

## Introduction

An upper-limb prosthesis is far less efficient than a normal arm because its movement is constrained to a few degrees of freedom, which cannot fully replace the complex motions critical for daily activities.^1,2^ The lack of dexterous hand function forces the prosthesis user to adopt compensatory motor strategies to accomplish tasks.^2,3,4^ This limitation is compounded by an absence of direct sensory feedback, resulting in the need for ongoing visual monitoring to guide the actions of the prosthesis.^5,6^ Technological developments attempt to address these challenges with multifunction prosthetic hands and methods of providing sensory feedback.^7,8,9^ However, no standard has been established to date for evaluating the improvements associated with these new techniques.^10,11^ It is important to assess whether the essential features of normal limb function such as visual attention, movement quality, and motor dexterity are restored by emerging technologies. Objective quantitative assessment of these features of human-prosthesis behavior could provide direction for device development, training, and treatment options.

Although rater-based methods to assess prosthesis function exist,^11,12^ movement quality and visual attention can be quantified more precisely with motion and eye tracking.^5,6^ However, comparison of outcomes across studies is limited by the wide variety of assessment tasks used.^13^ Task selection may be crucial to measured outcomes given that in-laboratory tasks that require only limited movements may not be generalizable to daily functional activities.^14,15^ Information is generally lacking on the intricate dependencies between upper-body movements and associated visuomotor behavior of prosthesis users when carrying out more complex goal-oriented tasks, because studies typically use seated tasks with objects placed immediately in front of the participant.^5,6,16,17^ For in-laboratory testing to be extrapolated to clinical real-world function, the testing paradigm should ideally represent behaviors that the prosthesis is to be used for in daily life.

To address this requirement, we developed 2 tasks that incorporated goal-directed object movements representative of daily activities^18^ and validated a method of quantifying angular joint kinematics, hand function movements, and eye gaze behavior.^19,20,21^ The purpose of the present study was to ascertain whether specifically designed tasks would identify different visuomotor adaptations by prosthesis users across tasks. We believe this comparison will provide insight into whether prosthesis users adapt to task challenges similarly across varying task requirements and thereby will address the unanswered questions: is task selection a factor in the compensation strategies chosen by prosthesis users, and does it complicate the measurement of outcomes for different interventions? We hypothesized that movement compensations would be affected by the placement of objects in the physical task setup, whereas visual adaptations would be more evident for a task with greater risks associated with grasp modulation.

## Methods

### Participants

This cross-sectional study was conducted in a single research center at the University of Alberta, Edmonton, Alberta, Canada. Prosthesis users were recruited from community prosthesis shops between January 1, 2016, and December 31, 2016, and were required to have a well-fitting socket and be able to grasp and move objects with their prosthesis. Individuals with normal arm function were recruited as the normative reference from October 1, 2015, to November 30, 2015. The study followed the Declaration of Helsinki guidelines^22^ and the Strengthening the Reporting of Observational Studies in Epidemiology (STROBE) reporting guideline. Approval for this study was granted by the University of Alberta Health Research Ethics Board, the Department of the Navy Human Research Protection Program, and the Space and Naval Warfare Systems Center Pacific (SSC-Pacific) Human Research Protection Office. Written informed consent was obtained from all participants.

### Functional Tasks and Data Collection

Participants performed 2 goal-oriented tasks.^18^ The cup transfer task (cup) challenged the ability of the participant to modulate grasp force while transferring compliant cups full of beads (to simulate water) over a low barrier across a table in front of the participant. The pasta box transfer task (box) required the participant to turn to the side to grasp a pasta box and then place it on shelves at varying heights in front and across the body of the participant. Although the pasta box has some inherent instability owing to its shifting contents, the task was assumed to be less risky because dropping or compressing the box would not result in spillage of contents.

Participants were requested to complete each task in a standardized sequence at a comfortable pace that allowed them to be as accurate as possible (as reported by Valevicius et al^18^) without spilling any beads for the cup task and without dropping the box or hitting the edges of the shelving for the box task. Tasks were performed using the prosthetic limb or, for participants with normal arm function, the right hand. Each prosthesis user attempted to achieve 10 error-free trials for each task, repeating the trial in case of an error but stopping if 5 consecutive errors were made to avoid frustration. Each participant with normal arm function performed 20 error-free trials for each task.

### Experimental Setup

Kinematic data were acquired at 120 Hz using a 12-camera motion capture system (Vicon Motion Systems Ltd). A synchronized head-mounted binocular eye tracker (Dikablis Professional 2.0; Ergoneers GmbH) captured simultaneous gaze behavior at 60 Hz. A kinematic model using cluster markers validated by Boser et al^19^ was used. Reflective markers were placed on the index finger and thumb, and marker clusters were affixed to upper body segments (pelvis, trunk, upper arms, forearms, hand, and head) or on analogous locations on the prosthesis. Additional markers were placed on the task-relevant items of the task setup, as was done by Valevicius et al.^18^

### Statistical Analysis

Kinematic and gaze-tracking trial data were segmented into reach, grasp, transport, and release phases and time-normalized using the procedures by Valevicius et al.^18^ Percentage of eye fixation on the upcoming target of action (current), hand or terminal device alone (hand), or holding the object in transport was calculated using the procedures described by Lavoie et al.^21^ The timing of visual fixation on the object and the visual disengagement from the object were quantified by a modified calculation of the eye arrival latency (EAL) and eye leaving latency (ELL), described by Lavoie et al,^21^ denoting the first arrival of the eye at and the first leaving of the eye from the grasp and release locations, compared with the start and end times of grasp and release (eFigure 1 in the Supplement). For each participant, the dependent measures for every trial and the mean across trials were calculated.

For the kinematic analysis, 3-dimensional joint angles were calculated according to the procedures by Boser et al^19^ and Valevicius et al.^20^ Range of motion (ROM) values (for trunk, shoulder, elbow, and wrist) for the entirety of every trial and the mean across trials were calculated. To quantify the differences in values between prosthesis users and those with normal arm function (ie, amount of compensation) and compare the values across the 2 tasks, we calculated the deviation values^23^ for phase duration and ROM as follows:

Deviation Value = (Prosthesis User Value − Mean Normative Value) × 10/SD in Normative Value,

where SD marks 1 SD. These values were plotted such that a deviation value of 0 indicates mean normative value, a deviation of 20 indicates mean normative value +2 SDs, and a deviation of −20 indicates mean normative value −2 SDs. According to the convention of defining the normal range by the 2-SD limits from the mean normative value,^23^ a deviation higher than 20 indicates compensatory movements and fewer than −20 indicates impairment or dysfunction.

Wilcoxon signed rank tests were performed to compare the 2 tasks within each group. Wilcoxon rank sum tests were conducted to compare the performance of each task between prosthesis users and participants with normal arm function. The statistical analysis used the JMP 13 software (SAS Institute Inc). Two-sided *P* < .05 was considered statistically significant. Data analysis was performed from December 3, 2018, to April 15, 2019.

## Results

### Participants

A convenience sample of 8 male prosthesis users with acquired amputation (mean [range] age, 45 [30-64] years) participated in the study. One prosthesis user was tested with 2 different devices, resulting in 9 total data sets (Table 1). Sixteen participants with normal arm function (8 men) participated, from whom we obtained normative reference values (mean [range] age, 26 [18-43] years; mean [range] height, 172.3 [158.0-186.0] cm; all right handed).

**Table 1.** Characteristics of the Prosthesis Users

Data Set	Prosthesis Side	Level of Amputation	Time Since Amputation, mo	Type of Prosthesis, End Effector	Prosthesis Use Frequency, h/d	AMULA Score
1	Left	Transhumeral	93	Body-powered, hook	14	16.7
2^a^	Left	Transhumeral	121	Myoelectric, hook	12	20.6
3^a^	Left	Transhumeral	121	Body-powered, hook	12	Not rated
4	Left	Transhumeral	31	Hybrid, hand	4	Not rated
5	Right	Transradial	62	Body-powered, hook	Not rated	16.7
6^b^	Right	Transradial	141	Body-powered, hook	14	17.8
7	Right	Transradial	115	Body-powered, hook	12	20.0
8	Left	Transradial	310	Body-powered, hook	8	21.7
9	Right	Transradial	336	Body-powered, hook	12	26.1

Abbreviation: AMULA, activities measure for upper limb amputees (score range: 0-40, with 0 indicating inability to perform and 40 indicating comparable performance to a sound limb^24^).

^a^ Data sets 2 and 3 are from the same participant using 2 different devices.

^b^ Bilateral, tested on right side.

### Task Performance and Duration

The mean (SD) total task time was significantly longer for prosthesis users (cup: 29.2 [16.4] seconds; box: 21.7 [7.4] seconds; *P* = .01) compared with participants with normal arm function (cup: 10.5 [1.2] seconds; box: 8.8 [1.2] seconds; *P* < .001), and the mean (SD) duration for the grasp and release phases were disproportionately prolonged (Table 2), significantly more so for the cup task than for the box task (grasp: 2.0 [2.3] seconds vs 0.9 [0.8] seconds; *P* < .001; release: 1.1 [0.6] seconds vs 0.7 [0.4] seconds; *P* < .001). Although the group comparison may have been unduly affected by 2 outlier participants with a transhumeral prosthesis (eFigure 2 in the Supplement), individual results all followed this pattern and, excluding these 2 outliers, maintained statistical significance (overall *P* = .01: grasp: 0.9 [0.3] seconds vs 0.5 [0.2] seconds; *P* = .02; release: 0.9 [0.5] seconds vs 0.6 [0.3] seconds; *P* = .03). Prosthesis users had errors on 17% of the attempted cup trials and 23% of the box trials, whereas participants with normal arm function had errors on 11% of the cup trials and 4% of the box trials.

**Table 2.** Comparison of Time, Eye-Fixation Metrics, and Joint Range of Motion Across the 2 Tasks Between Prosthesis Users and Participants With Normal Arm Function

Variable	Normative Values^a^			Prosthesis Users^a^			*P* Value Across Participant Groups	
	Cup Task	Box Task	*P* Value	Cup Task	Box Task	*P* Value	Cup Task	Box Task
Total time, s	10.5 (1.2)	8.8 (1.2)	<.001	29.2 (16.4)	21.7 (7.4)	.01	<.001	<.001
Phase duration, s								
Reach	0.7 (0.1)	0.6 (0.1)	<.001	1.5 (0.7)	1.4 (0.4)	.09	<.001	<.001
Grasp	0.2 (0.0)	0.2 (0.1)	.56	2.0 (2.3)	0.9 (0.8)	<.001	<.001	<.001
Transport	1.1 (0.1)	1.2 (0.1)	<.001	1.8 (0.5)	2.2 (0.7)	.05	<.001	<.001
Release	0.3 (0.1)	0.3 (0.1)	.11	1.1 (0.6)	0.7 (0.4)	<.001	<.001	<.001
**Eye Metrics**								
Current target, % fixation								
Reach	80.2 (9.8)	62.8 (11.0)	<.001	72.7 (12.7)	76.7 (10.3)	.65	.14	<.001
Transport	76.4 (9.1)	67.9 (5.3)	<.001	50.9 (11.1)	55.2 (10.5)	.16	<.001	<.001
Hand, % fixation								
Reach	1.1 (1.9)	0.1 (0.1)	<.001	10.2 (12.1)	2.2 (2.8)	<.001	<.001	<.001
Transport	7.5 (4.7)	6.8 (3.9)	.49	37.1 (9.7)	22.3 (7.6)	<.001	<.001	<.001
EAL, s								
Grasp	0.5 (0.1)	0.4 (0.1)	<.001	1.0 (0.2)	1.1 (0.4)	.57	<.001	<.001
Release	0.8 (0.1)	0.8 (0.1)	.07	0.9 (0.2)	1.2 (0.3)	<.001	.34	<.001
ELL, s								
Grasp	0.0 (0.1)	−0.1 (0.1)	.59	−0.8 (0.4)	−0.6 (0.5)	.42	<.001	<.001
Release	0.0 (0.1)	0.0 (0.1)	.97	−0.2 (0.2)	−0.7 (0.4)	<.001	<.001	<.001
**Kinematics, ROM Degrees**								
Trunk								
Flexion and extension	11.9 (4.6)	6.7 (1.7)	<.001	21.2 (3.7)	32.1 (10.7)	.01	<.001	<.001
Lateral bending	9.1 (2.7)	13.8 (3.1)	<.001	20.0 (6.8)	23.4 (7.9)	.09	<.001	<.001
Axial rotation	17.9 (3.4)	27.0 (3.0)	<.001	26.6 (5.7)	36.1 (8.8)	<.001	<.001	<.001
Shoulder								
Flexion and extension	78.7 (10.6)	88.0 (9.4)	<.001	41.0 (9.4)	51.3 (12.6)	.01	<.001	<.001
Abduction and adduction	43.8 (8.7)	32.5 (8.6)	<.001	32.3 (5.1)	40.5 (7.2)	.02	<.001	.02
Rotation	59.7 (14.9)	58.0 (8.0)	.78	35.5 (10.0)	50.6 (15.7)	.02	<.001	.20
Elbow, flexion and extension	87.6 (8.5)	94.4 (10.6)	<.001	32.4 (23.8)	51.0 (27.2)	.02	<.001	<.001
Wrist, flexion and extension	87.4 (12.7)	39.6 (8.8)	<.001	4.4 (1.8)	8.4 (5.5)	.01	<.001	<.001
Wrist, ulnar/radial, flexion	43.1 (7.5)	36.0 (4.3)	<.001	4.7 (3.8)	6.2 (3.8)	.16	<.001	<.001

Abbreviations: EAL, eye arrival latency; ELL, eye leaving latency; ROM, range of motion.

^a^ Values are the mean (SD) of all trials.

### Eye Gaze Behavior

**Figure 1.**
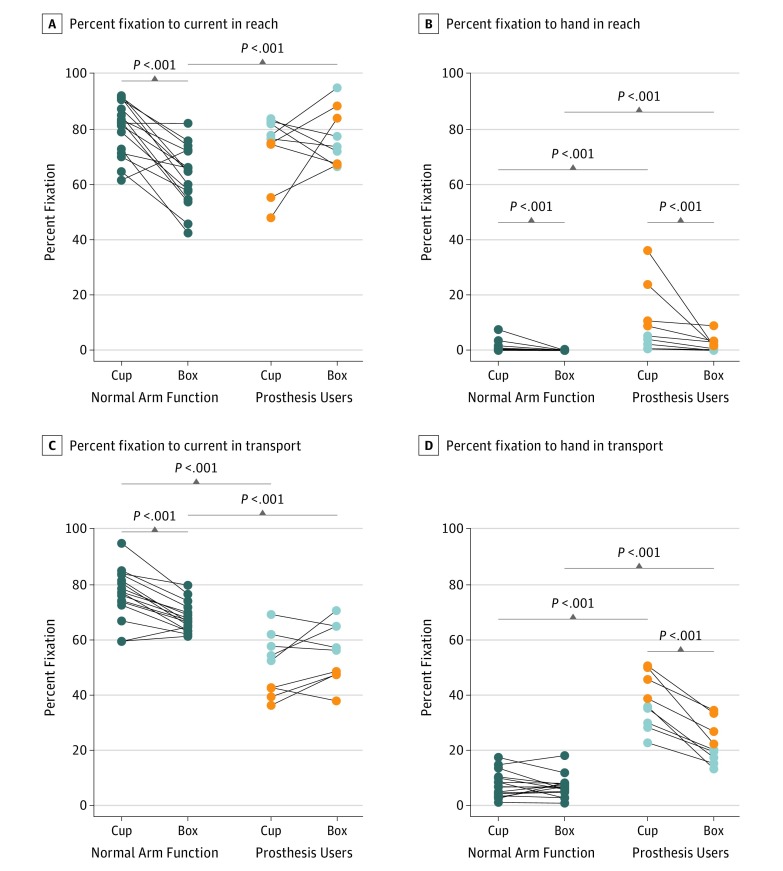
Percentage of Eye Fixation to Current and Hand Areas of Interest During Reach and Transport Phases For prosthesis users, light gray circles represent transradial and orange circles represent transhumeral prosthesis users. Cup refers to the cup transfer task, and box refers to the pasta box transfer task. Wilcoxon signed rank test was performed to compare the 2 tasks, and Wilcoxon rank sum test was used to compare participants with normal arm function with prosthesis users (details in Table 2).

Percentage of eye fixation on the current target indicated the relative duration of look-ahead fixation in reach and transport phases, which was not significantly different across tasks for prosthesis users (Table 2 and Figure 1A-C). This finding was in contrast to the behavior of participants with normal arm function, which displayed greater mean (SD) fixation on current target in both reach and transport phases for the cup task (reach: 80.2% [9.8%]; transport: 76.4% [9.1%]) compared with the box task (reach: 62.8% [11.0%]; transport: 67.9% [5.3%]). Compared with participants with normal arm function, prosthesis users had significantly less mean (SD) fixation on the current target during the transport phase for both tasks (cup: 76.4% [9.1%] vs 50.9% [11.1%], *P* < .001; box: 67.9% [5.3%] vs 55.2% [10.5%], *P* < .001), but fixation on the current target during the reach phase was either not significantly different (cup: 80.2% [9.8%] vs 72.7% [12.7%], *P* = .14) or significantly higher (box: 62.8% [11.0%] vs 76.7% [10.3%], *P* < .001).

The prosthesis users fixated on the hand in reach for a significantly greater mean (SD) percentage of both reach (10.2% [12.1%] vs 2.2% [2.8%], *P* < .001) and transport (37.1% [9.7%] vs 22.3% [7.6%], *P* < .001) phases for the cup task compared with the box task (Table 2 and Figure 1B-D) and compared with participants with normal arm function. The largest magnitude was for cup transport (Figure 1D), with a mean increase of 30% fixation on the hand in reach compared with participants with normal arm function. Participants with normal arm function had significantly greater mean (SD) fixation on the hand in reach but not transport for the cup task (reach: 1.1% [1.9%]; transport: 7.5% [4.7%]) compared with the box task (reach: 0.1% [0.1%]; transport: 6.8% [3.9%]).

**Figure 2.**
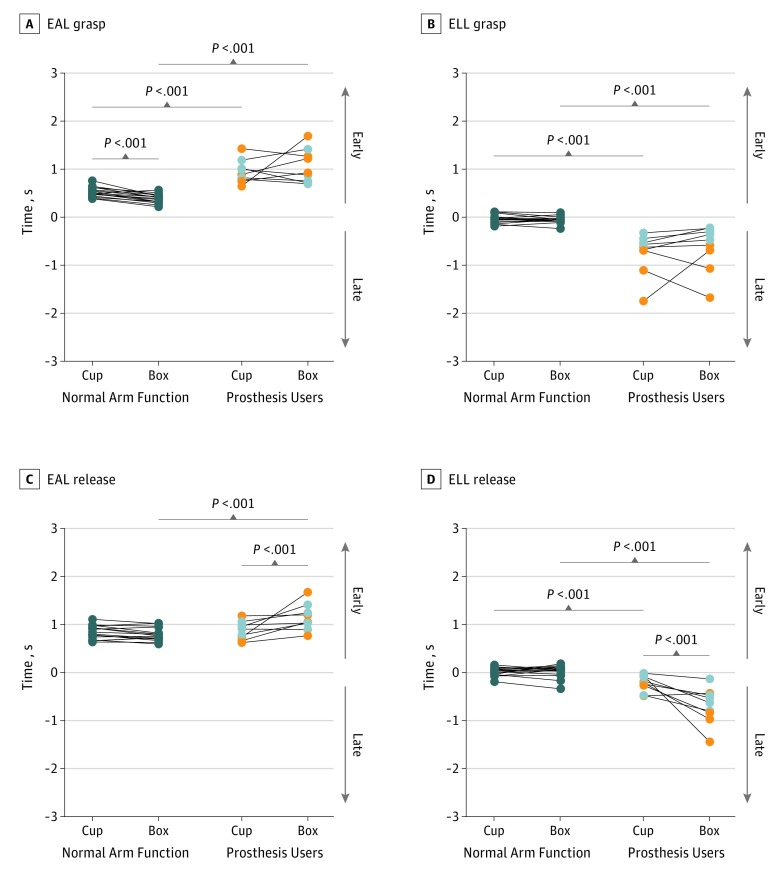
Comparison of Eye Arrival Latency (EAL) and Eye Leaving Latency (ELL) for Grasp and Release For prosthesis users, light gray circles represent transradial and orange circles represent transhumeral prosthesis users. Cup refers to the cup transfer task, and box refers to the pasta box transfer task. Wilcoxon signed rank test was performed to compare the 2 tasks, and Wilcoxon rank sum test was used to compare participants with normal arm function with prosthesis users (details in Table 2).

Prosthesis user eye latency results reflected significant task differences during release of the object but not during grasp (Table 2 and Figure 2). For the box task, compared with the cup task, prosthesis users fixated on the release target significantly earlier (mean [SD], 1.2 [0.3] seconds vs 0.9 [0.2] seconds; *P* < .001) (Figure 2C) and the gaze lingered significantly longer after release (mean [SD], −0.7 [0.4] seconds vs −0.2 [0.2] seconds; *P* < .001) (Figure 2D). Normative mean (SD) EAL and ELL times were not significantly different across tasks, with the exception of the EAL grasp (Figure 2A), which showed a minor but significant difference for earlier arrival in the cup task (0.5 [0.1] seconds vs 0.4 [0.1] seconds for the box task; *P* < .001). Prosthesis users, compared with participants with normal arm function, had significantly longer mean (SD) EAL and ELL times for all values (cup task EAL grasp: 1.0 [0.2] seconds vs 0.5 [0.1] seconds and ELL grasp: −0.8 [0.4] second vs 0.0 [0.1] second; release: −0.2 [0.2] second vs 0.0 [0.1] second. Box task EAL grasp: 1.1 [0.4] seconds vs 0.4 [0.1] second and ELL grasp: −0.6 [0.5] second vs −0.1 [0.1] second; release −0.7 [0.4] second vs 0.0 [0.1] seconds; all *P* < .001), except EAL release during the cup task (0.9 [0.2] second vs 0.8 [0.1] second; *P* = .34) (Figure 2C).

### Kinematic Results

**Figure 3.**
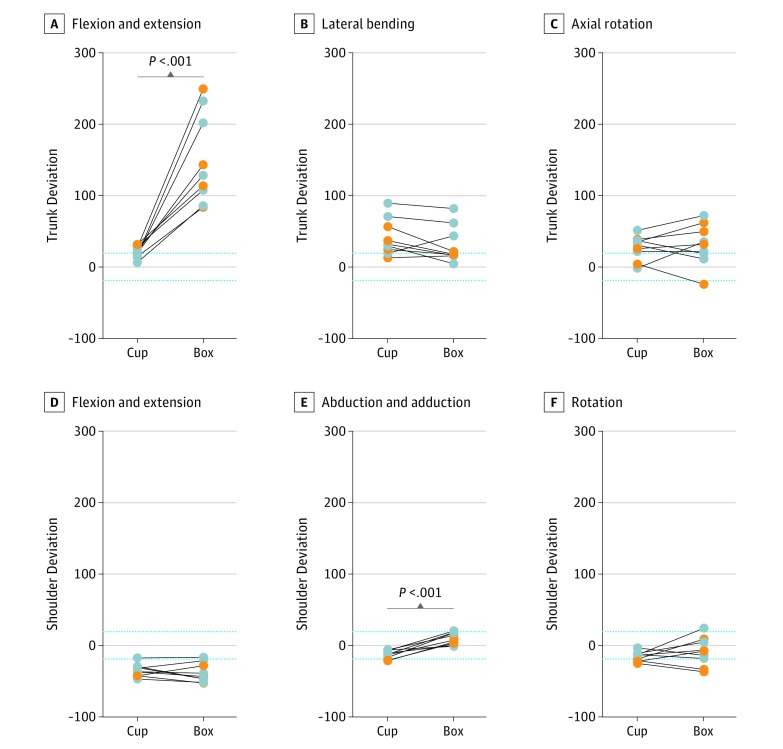
Trunk and Shoulder Kinematic Deviation Values for Prosthesis Users Deviation of the prosthesis user values from participants with normal arm function are plotted for the 3 degrees of freedom of the trunk and shoulder, compared across tasks. Deviation of 0 indicates mean normal arm function value, with 2-SD limits indicated by dotted lines, such that deviation ranging from −20 to 20 indicates the normal range. Gray circles represent transradial and orange circles represent transhumeral prosthesis users. Cup refers to the cup transfer task, and box refers to the pasta box transfer task.

In addition to absolute ROM values (Table 2), the deviation of prosthesis user movement from the values of participants with normal arm function was examined to identify which task required the most excessive compensations (Figure 3).

In general, mean (SD) trunk ROM was significantly greater for prosthesis users (cup: 21.2 [3.7] degrees; box: 32.1 [10.7] degrees; *P* = .01) compared with participants with normal arm function (cup: 11.9 [4.6] degrees; box: 6.7 [1.7] degrees; *P* < .001) across both tasks and all planes of movement (Table 2). However, the prosthesis user deviation values revealed that the box task involved significantly greater trunk flexion and extension deviation compared with the cup task (mean [SD], 32.1 [10.7] degrees vs 21.2 [3.7] degrees; *P* = .01) (Figure 3), whereas the data of participants with normal arm function showed more trunk flexion and extension in the cup task compared with the box task (mean [SD], 11.9 [4.6] vs 6.7 [1.7]; *P* < .001) (Table 2). For trunk lateral bending and axial rotation, no significant differences were observed between the prosthesis user deviation values across tasks.

Prosthesis users displayed significantly more shoulder ROM in all 3 planes for the box task compared with the cup task (mean [SD] flexion and extension; 51.3 [12.6] degrees vs 41.0 [9.4] degrees, *P* = .01; abduction and adduction: 40.5 [7.2] degrees vs 32.3 [5.1] degrees, *P* = .02; rotation: 50.6 [15.7] degrees vs 35.5 [10.0] degrees, *P* = .02) (Table 2). In contrast, the normal arm function group used significantly more shoulder flexion and extension for the box task (mean [SD] ROM, 88.0 [9.4] degrees) and more shoulder abduction and adduction ROM for the cup task (43.8 [8.7] degrees), with no difference in shoulder rotation (cup: 59.7 [14.9] degrees vs box: 58.0 [8.0] degrees; *P* = .78). Among prosthesis users, shoulder abduction and adduction for the box task was the only shoulder movement that was greater than the normative values (mean [SD], 40.5 [7.2] degrees vs 32.5 [8.6] degrees; *P* = .02), with almost all other values for prosthesis users significantly below those for participants with normal arm function (Table 2). Deviation values supported the finding that the box task required a significantly greater abduction and adduction range compared with the cup task, although most values were within the user range of participants with normal arm function (Figure 3).

Elbow flexion and extension ROM for prosthesis users was greater for the box task compared with the cup task (mean [SD] ROM, 51.0 [27.2] degrees vs 32.4 [23.8] degrees; *P* = .02), although ROM was significantly reduced for prosthesis users across both tasks compared with participants with normal arm function (mean [SD] ROM, 94.4 [10.6] degrees vs 87.6 [8.5] degrees; *P* < .001) (Table 2). Wrist motion was not relevant because all prosthesis users had static wrist components that they locked in a preferred position at the start of each task, and any recorded motion was associated with compliance at the mechanical coupling.

## Discussion

### Visual Attention Association With Task Demands

The prosthesis users visually fixated on their hand more than participants with normal arm function did for both tasks; however, the cup task required more visual attention to the hand compared with the box task. This allocation of visual attention likely represents a cautious strategy by prosthesis users to approach the cup without knocking it over and monitor the compliance of the cup during transport to avoid spilling the contents. The challenge of handling the cups was reinforced by the greater prolongation of grasp and release times, which was not observed in the performance by participants with normal arm function. In the cup task, the placement of the cups in front of the participants with normal arm function was found to be a factor in increasing visual fixation compared with the box task; however, these individuals did not visually monitor the transport of the compliant cups as much as the prosthesis users did. Specifically, prosthesis users, compared with participants with normal arm function, showed greater fixation on the hand for cup transport than for the box task at the expense of reduced ability to look ahead. Previous studies of gaze behavior during prosthesis use have shown similar findings of increased fixation on the hand and decreased ability to look ahead during object interaction tasks using both noncompliant^6,25^ and compressible^5,16^ objects. The reduced ability to switch visual fixation to the next target placement may impair motor planning given that visual fixation is a key requirement for coordinated object manipulation^26^ and is a likely factor in the prolonged movement times commonly observed in prosthesis use.

Substantial differences were observed in how the prosthesis users adapted to moving objects through a larger workspace, as required for the box task. Individuals with normal arm function required only a momentary glance to locate the object; their arm was then guided by intact proprioception and tactile sensory feedback to fluidly grasp and move the object.^26^ Despite this capability, gaze fixation patterns in individuals with normal arm function were shown to be affected by task and context.^27^ Results of the present study were consistent with those of a previous study that showed less fixation for the first reach of the pasta box task, which required the participant to turn and fixate outside of the initial field of view.^21^ In contrast, when prosthesis users were challenged by an object outside of their immediate field of view, they increased fixation on the pasta box when reaching for it.

Given the nature of prosthesis control, to successfully interact with the object, the prosthesis users likely had to move their body to face the box to physically grasp it and therefore had more opportunity to visually fixate on the object. Essentially, the prosthesis users may be operating in such a way that their vision relies on body orientation movements rather than leading their body movements, as is expected with normal visuomotor sequencing.^21^ The prosthesis users’ gaze also lingered significantly longer on the pasta box after release than for the cups, possibly owing to the inherent instability of the narrow base of the box and the desire to ensure the box did not fall over after release, which was noted as a common error.

Most studies of visual fixation in prosthesis users use a tabletop task setup in front of the participant.^5,6,16,25^ Results of this study suggest that the task design changes the visuomotor adaptations observed and that interacting with objects solely on a table in front of the participant may limit the generalizability of the findings to other functional tasks.

### Different Movement Strategies Between Tasks

Normal hand dexterity and arm function allowed participants with normal arm function to accomplish the tasks with minimal trunk movement, relying on wrist, elbow, and shoulder motion similar to that cited for common daily tasks.^28^ Prosthesis users used excessive trunk motion as the main movement compensation to accomplish both tasks. However, the trunk movement strategies used by the prosthesis users across tasks were reversed compared with strategies of participants with normal arm function, with greater trunk flexion and extension for the box task compared with the cup task. The need for different trunk compensations across tasks was likely due to the lack of wrist motion for positioning the terminal device to reach different positions on a table compared with the different heights on a shelf. Wrist motion is integral for object interaction tasks, and prostheses that do not provide wrist motion have been shown to be associated with compensatory changes at the elbow, shoulder, and trunk.^1,2,3,4^ The tasks differentially challenged wrist motion in both the sagittal and frontal planes by having different grasp and placement requirements for the objects, which affected the trunk compensation strategy.

The role of shoulder movements as compensation for prosthesis users is controversial, as some studies have reported increased shoulder movements,^3,29^ whereas others reported decreased shoulder movements.^1,17^ In this study, prosthesis users showed a significant reduction in all shoulder motions, compared with normal movement patterns, for the cup task. For the box task, other than shoulder abduction, the prosthesis users applied similar or less shoulder motion as the participants with normal arm function yet still required greater trunk compensation, indicating the increased kinematic demands of the box task.

These differences highlight the importance of using tasks that challenge a workspace outside of the immediate frontal sphere of the prosthesis user to fully assess the functional limitations, a notable deficit of the current assessments.^11^ The imposed shoulder restriction could be from the socket fit, harnessing, or the need to keep the prosthesis and residual limb in a position in front of the body for control, but it was seen across all users. Prosthesis users, thus, experience not only impairments at the wrist and hand owing to anatomical loss but also additional constraints at the elbow and shoulder owing to the prosthesis.

### Compensation Patterns Among Prosthesis Users

Despite the heterogeneity of the prosthesis users, compensation trends generally followed the same pattern, with the most skilled users occasionally reaching the range of normal movement. These findings are consistent with those of Metzger et al,^3^ who found similar compensation patterns across a sample of users with transhumeral, transradial, myoelectric, or body-powered prostheses performing complex tasks. The striking similarity of the compensation patterns, which may have been enforced by the strict task design, suggests that prosthesis users may have a common method of functioning for object interaction tasks, irrespective of the level of impairment or device. This theory would be consistent with the known central neural adaptation and maladaptive plasticity occurring after amputation, which has been postulated to impair motor learning and result in heightened visuospatial needs.^30^ Participants with transhumeral prostheses may have changed some of the trends in that clear outliers were observed for several of the visual fixation results. However, the transhumeral prosthesis users were nearly indistinguishable from the transradial prosthesis users in trunk and shoulder kinematic compensations. Future studies should identify whether divergent trends emerge on the basis of amputation level, skill level, or training and whether the changes in visuomotor adaptations can be altered with improved prosthetic dexterity or sensory feedback.

Understanding the deviation from normal performance (rather than the absolute value) may help in identifying the source of the movement problem for selecting rehabilitation training interventions. The impairment of shoulder motion seen in this study might be addressed with prosthetic design or a training intervention to restore normal motion and reduce truncal compensation. One goal of rehabilitation is to enhance motor learning and reduce unnecessary compensations to bring movement patterns as close as possible to normal movement patterns^17^; however, some have argued that changed motor patterns may be adaptive in some cases and should be optimized rather than corrected.^31^ In either case, quantification of visuomotor compensations could be the basis for planning interventions that facilitate the ability of upper-limb prosthesis users to adapt to novel task environments through training and optimization.^17,32^ Findings of this study appear to reinforce the importance of task selection to challenge the visuomotor adaptation strategies of the prosthesis user across more interaction zones during treatment and assessment.

### Limitations

This study has several limitations. The tasks did not require bimanual coordination, which is an important goal of prosthesis use, but would have been a factor in the visuomotor behavior given the known dependencies of prosthesis users on the intact arm.^14^ The inferences of the study were limited by the size of the prosthesis user sample. In addition, the prosthesis users were generally older than the participants with normal arm function and were all male, and there was a predominant proportion of body-powered transradial prosthesis users. Our assessment quantified aspects of human-prosthesis interaction that cannot necessarily be ascertained by clinical observation skills alone but required instrumentation and technology not commonly available in the clinic. In the clinical realm, simplifying the assessment process may be desired rather than increasing the technological and administrative burden. However, in doing so, we risk losing information that may be most relevant to the goal of expanding prosthesis function.

## Conclusions

In this study, visuomotor adaptations by a range of prosthesis users were quantified across 2 tasks that were designed to challenge different functional requirements. The specific task used for analysis was associated with different visuomotor compensation strategies and thereby altered the findings between tasks. The cup task was suitable to evaluate grasping skills and compensatory gaze movements, and the box task brought out specific kinematic compensatory strategies that were required not only to move the object successfully but also to adapt visual requirements. The similarities among prosthesis users suggest a common motor planning approach to tasks that is not easily adapted to varying task demands. Assessment tasks should, therefore, challenge the prosthesis user in a workspace that is closer to daily functional requirements so that the evaluator may fully appreciate the limitations and potential advantages of upper-limb prosthesis interventions.

## Notes

Supplement. **eFigure 1.** Definitions of Eye Latency Measures **eFigure 2.** Phase Duration Deviation Values for Prosthesis Users Compared Across Tasks
