# Supplementary material for: Quantitative Eye Gaze and Movement Differences in Visuomotor Adaptations to Varying Task Demands Among Upper-Extremity Prosthesis Users
Source: JAMA Netw Open. 2019 Sep 13;2(9):e1911197. doi: 10.1001/jamanetworkopen.2019.11197 (PMC6745056; doi:10.1001/jamanetworkopen.2019.11197)
Supplement: Supplement. — eFigure 1. Definitions of Eye Latency Measures eFigure 2. Phase Duration Deviation Values for Prosthesis Users Compared Across Tasks [file jamanetwopen-2-e1911197-s001.pdf]

# Supplementary Online Content

Hebert JS, Boser QA, Valevicius AM, et al. Quantitative eye gaze and movement differences in visuomotor adaptations to varying task demands among upper-extremity prosthesis users. *JAMA Netw Open*. 2019;2(9):e1911197. doi:10.1001/jamanetworkopen.2019.11197

**eFigure 1.** Definitions of Eye Latency Measures

**eFigure 2.** Phase Duration Deviation Values for Prosthesis Users Compared Across Tasks

This supplementary material has been provided by the authors to give readers additional information about their work.

© 2019 Hebert JS et al. *JAMA Network Open*

**eFigure 1.** Definitions of Eye Latency Measures

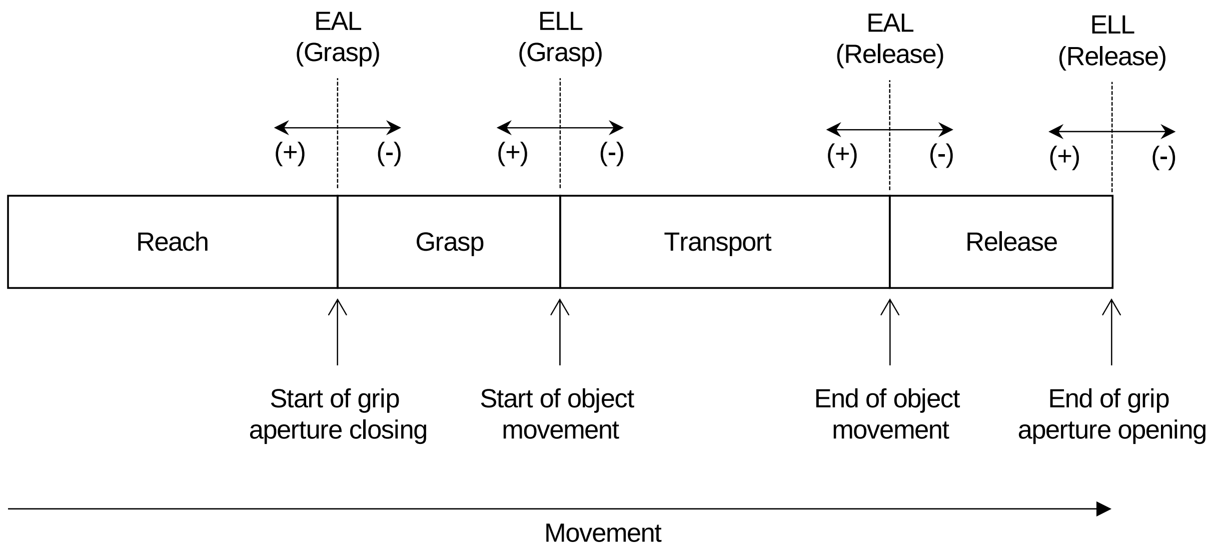


Eye Arrival Latency (EAL) and Eye Leaving Latency (ELL) at grasp were bound to the start and end of the grasp phase and denoted the first arrival of the eye to and the first leaving of the eye from the grasp location, respectively. EAL (Grasp) was defined as Grasp start time minus the time of eye arrival at the cup location. Thus, EALg was positive if the eyes began fixating on the object before Grasp began, and negative if the eyes began fixating on the object after Grasp began. ELL (Grasp) was defined as Transport start time minus the time of the eye first looking away from grasp, and was positive if the eye looked away from the grasp area prior to the end of grasp, and negative if the eye lingered on the object after it started moving in transport. Similar values bound to the timing of release of the object at the designated target were defined by EAL and ELL at release. EAL (Release) was calculated as Transport end time minus the time of eye arrival at the drop off location. ELL (Release) was defined as Release end time minus the time of the eye leaving the drop-off location. This ELL (Release) value was positive if the eyes ended their fixation on the target before Release ended, and negative if the eyes ended their fixation on the target after Release ended.

© 2019 Hebert JS et al. *JAMA Network Open*

**eFigure 2.** Phase Duration Deviation Values for Prosthesis Users Compared Across Tasks

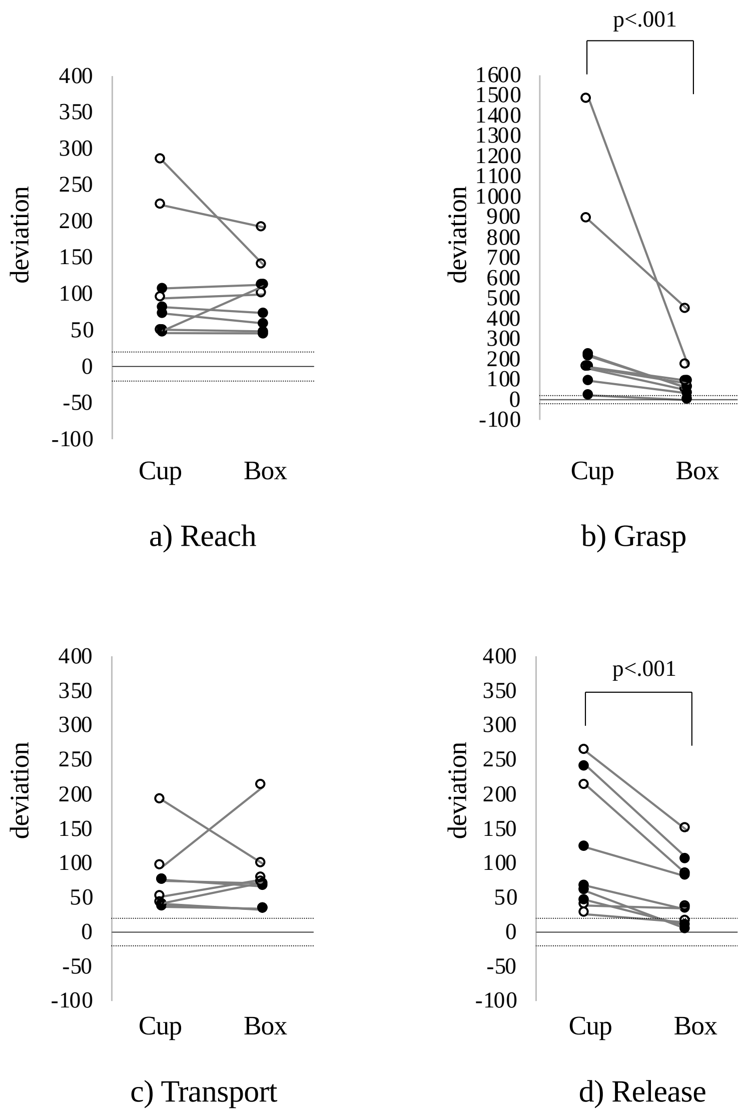


Deviation of the prosthesis user values from mean normative values are plotted for each phase of movement, and compared across tasks. Solid line at deviation 0 indicates mean normative value, with +/- 2SD limits indicted by dotted lines. There was significantly longer prolongation of grasp (b) and release phases (d) for the Cup Task compared to the Box Task, with consistent trends across all participants for these two phases. For reach (a) and transport (c), all prosthesis user deviation values were prolonged outside the range of normal values but with no task differences.

© 2019 Hebert JS et al. *JAMA Network Open*
